# CAS-YOLO: A lightweight and efficient object detector for infrared UAV imagery

**DOI:** 10.1371/journal.pone.0359167

**Published:** 2026-09-25

**Authors:** Jiayin Liu, Yuyuan Shen, Shujun Ji, Zhi Liu, Huilin Jiang

**Affiliations:** 1 College of Optoelectronic Engineering, Changchun University of Science and Technology, Changchun, Jilin, China; 2 Optronics, Aviation Industry Corporation of China, Luoyang, Henan, China; Beijing Institute of Technology, CHINA

## Abstract

Infrared target detection is crucial for unmanned aerial vehicle (UAV) applications in civilian low-visibility environments, including urban nighttime monitoring, emergency rescue, and infrastructure inspection. However, thermal imaging blur, complex backgrounds, and extreme target aspect ratios pose significant challenges to thermal infrared target detection. Moreover, conventional real-time detectors, constrained by the limited edge computing resources of UAVs, typically rely on inefficient square convolutions and computationally expensive attention mechanisms. To address these issues, we propose CAS-YOLO, an efficient and lightweight detector specifically tailored for civilian thermal infrared UAV imagery. Building upon YOLOv10n, CAS-YOLO selectively deploys Large Strip Convolution (LSC) at intermediate backbone stages to enhance the directional structures of elongated targets while reducing interference from surrounding thermal clutter. Circulant Contextual Attention (CCA) is placed at the terminal low-resolution stage to aggregate long-range contextual information without applying global modeling to high-resolution feature maps. StarReLU is applied throughout the basic convolutional units as a lightweight nonlinear transformation for weak-texture infrared features. Experiments on HIT-UAV, together with auxiliary cross-domain evaluation on FLIR, show that CAS-YOLO improves detection accuracy within a YOLOv10n-based lightweight framework. The ablation studies evaluate the individual effects of the three components and the complementary effects of LSC and CCA. This study is strictly limited to civilian applications in public safety, traffic management, and autonomous driving assistance.

## 1. Introduction

In recent years, unmanned aerial vehicles (UAVs) equipped with thermal infrared imaging payloads have played an increasingly pivotal role in civilian low-visibility mission scenarios, including urban nighttime monitoring, disaster emergency rescue, infrastructure inspection, and autonomous driving assistance. Unlike visible-light imaging, thermal infrared imaging technology is unconstrained by illumination conditions, enabling target detection through thermal radiation penetration of haze and dark environments. Consequently, constructing efficient and accurate infrared UAV target detection systems has become a significant research direction in the computer vision community. However, achieving simultaneous high detection accuracy and real-time performance in practical engineering deployments remains a formidable challenge, constrained by stringent onboard edge computing limitations, power budget restrictions, and the inherent physical imaging characteristics of infrared imagery.

Currently, real-time deep learning-based object detectors—particularly the YOLO family—have achieved remarkable progress in general vision tasks, yet they exhibit pronounced performance bottlenecks when confronted with complex scenarios from infrared UAV perspectives (as typified by the HIT-UAV dataset). Infrared images generally lack rich color and textural details and are susceptible to thermal imaging blur induced by atmospheric disturbances and sensor noise. Such complex background clutter environments demand robust global contextual perception capabilities from detection models to effectively separate faint targets from chaotic backgrounds. However, while standard self-attention mechanisms can provide global receptive fields, their computational complexity scales quadratically as $O(N^{\text{2}})$ with feature map dimensions, resulting in prohibitive computational overhead that precludes deployment on resource-constrained UAV edge computing platforms.

Beyond the difficulties of global feature modeling, the geometric morphological characteristics of targets themselves further exacerbate detection challenges. Under UAV nadir viewing perspectives, traffic participants such as vehicles and pedestrians often exhibit highly elongated structural configurations, demonstrating significant anisotropy and extreme aspect ratios. Conventional detection networks predominantly employ square convolution windows for feature extraction; this isotropic receptive field design inevitably introduces substantial irrelevant neighborhood background information when processing high-aspect-ratio targets, severely degrading the model's feature discriminability and localization precision. Moreover, the complex and variable feature distributions of infrared targets impose higher requirements on the underlying nonlinear fitting capabilities of networks, rendering the trade-off between feature expressiveness and computational cost increasingly acute. Lightweight networks, in pursuit of inference efficiency at the edge, typically favor traditional activation functions with low computational overhead (e.g., ReLU). However, such functions are essentially piecewise linear mappings that struggle to establish deep nonlinear feature correlations when addressing complex thermal infrared characteristics, thereby limiting network expressiveness. Blindly introducing high-complexity activation functions to compensate for this deficiency would precipitate a steep surge in FLOPs, ultimately compromising overall real-time inference performance.

To address these technical challenges, this paper proposes CAS-YOLO, a lightweight object detection framework tailored to civilian infrared UAV scenarios. Built upon YOLOv10n, CAS-YOLO organizes three efficient components according to the characteristics of infrared imagery and the computational roles of different network stages. LSC [1] is selectively placed at intermediate backbone stages to enhance anisotropic structures while limiting thermal-background interference. CCA [2] is applied at the terminal low-resolution stage to aggregate long-range context without performing global modeling on high-resolution feature maps. StarReLU [3] is used in the basic convolutional units to provide a lightweight nonlinear transformation. This arrangement forms an ordered pathway from local directional structure enhancement to deep global context aggregation.

The principal contributions of this work are summarized as follows. We develop CAS-YOLO, a lightweight detection framework based on YOLOv10n and tailored to the characteristics of infrared UAV imagery, with particular emphasis on elongated-target representation, global contextual modeling, and computational efficiency. To accommodate the prevalence of small and high-aspect-ratio targets, LSC is selectively deployed at intermediate backbone stages to enhance anisotropic structural features while suppressing irrelevant thermal-background interference. CCA is placed at the terminal low-resolution stage to model global long-range dependencies at O(NlogN) computational complexity, thereby establishing a sequential pathway from local directional structure enhancement to deep global context aggregation. We further employ StarReLU in the basic convolutional units to improve nonlinear feature representation for weak-texture and low-contrast infrared targets with limited additional computational overhead. CAS-YOLO is systematically evaluated on the publicly available HIT-UAV infrared UAV dataset, with the ground-level FLIR dataset used for auxiliary cross-domain evaluation. The comparative experiments demonstrate improved detection accuracy under the reported settings, while the ablation studies verify the individual effects of the three components and the complementary effects of LSC and CCA.

## 2. Related works

### 2.1. Wide-field-of-view remote sensing and infrared target detection

With the development of deep learning, remote-sensing object detection has gradually shifted from handcrafted features to end-to-end detection frameworks. Wide-field-of-view remote-sensing images commonly contain substantial variations in target scale, orientation, and aspect ratio, making receptive-field expansion and geometric representation important research directions. LSKNet employs large-kernel convolution and spatial selection to adapt contextual information to different targets [4], while PKINet uses parallel multi-scale square kernels to establish feature associations across different spatial ranges [5]. Oriented R-CNN further improves the localization of objects with substantial orientation and aspect-ratio variations through optimized target representation [6]. These studies provide important foundations for multi-scale and oriented target detection in complex remote-sensing scenes.

When extending these methods to infrared UAV imagery, the detection problem becomes more challenging. Infrared images lack rich color and texture information, while small and elongated targets are easily obscured by weak contrast, imaging blur, and thermal-background clutter. The UAV nadir viewpoint further increases target-scale variation and geometric anisotropy, whereas limited onboard computing resources restrict the use of complex detection architectures. Recent lightweight YOLO variants have therefore been developed specifically for infrared UAV detection. DAN-YOLO-L enhances multi-scale feature extraction and small-target localization while reducing model redundancy through structured pruning [7]. DNR-YOLO addresses feature attenuation and boundary uncertainty through lightweight feature fusion and localization refinement [8]. CHT-YOLO combines dynamic sparse perception with task-decoupled alignment to improve the representation of weak infrared targets [9].

These methods demonstrate the effectiveness of task-specific enhancement and lightweight design for infrared UAV detection. However, existing approaches mainly emphasize small-target recovery, multi-scale fusion, or model compression, while the directional structures of elongated targets and long-range thermal-background dependencies are often addressed separately. Moreover, directly applying square large-kernel or parallel convolutional structures may introduce irrelevant surrounding information and additional computational costs. Therefore, infrared UAV detection requires a lightweight architecture that coordinates directional structural extraction and global-context modeling according to the spatial resolution and computational characteristics of different network stages.

### 2.2. Attention mechanisms in visual models

Self-attention mechanisms have been extensively incorporated into visual networks to enhance feature representation capabilities, owing to their capacity for capturing global-range dependency relationships. However, the computational complexity of standard self-attention mechanisms scales quadratically as $O\left(N^{\text{2}}\right)$ with sequence length, incurring prohibitive computational costs when processing high-resolution remote sensing imagery. To alleviate this bottleneck, researchers have conducted extensive investigations into improving self-attention efficiency. For example, Swin Transformer significantly reduces computational burden by restricting attention within non-overlapping local windows [10]; PVT adopts a strategy of downsampling keys and values to compress computational complexity [11]; methods such as DAT and BiFormer propose input-adaptive sparse attention patterns and bi-level routing attention mechanisms, respectively, to dynamically determine regions of interest [12,13].

Despite the computational efficiency improvements achieved by these modified approaches, their designs still possess fundamental limitations. These hand-crafted sparse or local patterns essentially impose external structural constraints on self-attention mechanisms, inevitably compromising the model's long-range global modeling capacity and scalability. In civilian infrared scenes with cluttered backgrounds, the absence of adequate global perception often leads to confusion between targets and background. Therefore, how to break through the limitations of hand-crafted patterns while preserving the expressive power of standard self-attention, achieve substantial reduction in computational complexity, and thereby attain more flexible collaborative modeling between global semantic information and computational efficiency, remains a critical problem awaiting in-depth investigation.

### 2.3. Activation functions and nonlinear feature representations

The nonlinear expressive capability of features plays a critical role in object detection under complex scenes. In lightweight detection networks pursuing inference speed, ReLU has become the default choice of activation function due to its computational simplicity and ability to alleviate gradient vanishing. However, infrared images often lack salient textural cues, and target feature distributions exhibit high complexity; relying solely on simple elementary activation functions hardly satisfies the feature selectivity requirements of deep networks.

ReLU, widely adopted in existing lightweight networks, is essentially a piecewise linear function. Despite its high computational efficiency, this simple linear mapping struggles to capture complex nonlinear feature correlations among pixels when processing faint and intricate feature mappings in infrared images. Some studies have attempted to introduce more complex activation functions to enhance network expressiveness, yet this typically comes at the cost of significantly increased computational overhead (FLOPs), degrading real-time inference speed on edge devices. Therefore, without exceeding the computational budget of lightweight networks (e.g., introducing only simple squaring and multiplication operations), how to select an efficient nonlinear transformation capable of adapting to data distributions, stabilizing gradient propagation, and possessing stronger nonlinear fitting capability remains another critical challenge for further improving the performance of lightweight infrared UAV target detection models.

### 2.4. Recent advances in UAV-enabled intelligent systems

Recent advances have transformed UAVs from conventional aerial imaging platforms into intelligent systems integrating autonomous navigation, wireless communication, and scene perception. Eskandari et al. developed an end-to-end trajectory generation framework based on visual generative adversarial networks, which uses bird’s-eye-view imagery to generate UAV trajectories satisfying obstacle-avoidance and kinodynamic constraints for RIS-assisted energy-efficient vehicular networks [14]. Mustari et al. investigated cooperative THz communication for UAVs in 6G and beyond, demonstrating the potential of flying ad hoc networks to support high-capacity wireless connectivity [15]. Obaid et al. combined UAV video with deep learning-based object detection and tracking to automate the estimation of turning movement rates at multilane roundabouts [16]. These studies illustrate the expanding role of UAVs in navigation, communication, and transportation analysis, while imposing increasing requirements on environmental perception, real-time processing, and deployment feasibility. Within this broader development, lightweight infrared object detection under low-visibility conditions and complex thermal backgrounds represents an important component of all-weather UAV perception and constitutes the specific focus of CAS-YOLO.

## 3. Methods

This article does not contain any studies with human participants or animals performed by any of the authors.

This section describes the proposed CAS-YOLO architecture. Section 3.1 presents the YOLOv10n-based network and explains the stage-specific deployment and ordered interaction of LSC, CCA, and StarReLU. Section 3.2 describes how LSC uses sequential orthogonal strip convolutions to enhance anisotropic structures at intermediate backbone stages. Section 3.3 presents CCA and its application to global context aggregation at the terminal low-resolution stage. Section 3.4 describes the use of StarReLU in the basic convolutional units as a lightweight nonlinear transformation for infrared features.

### 3.1. Network overview

**Fig 1:**
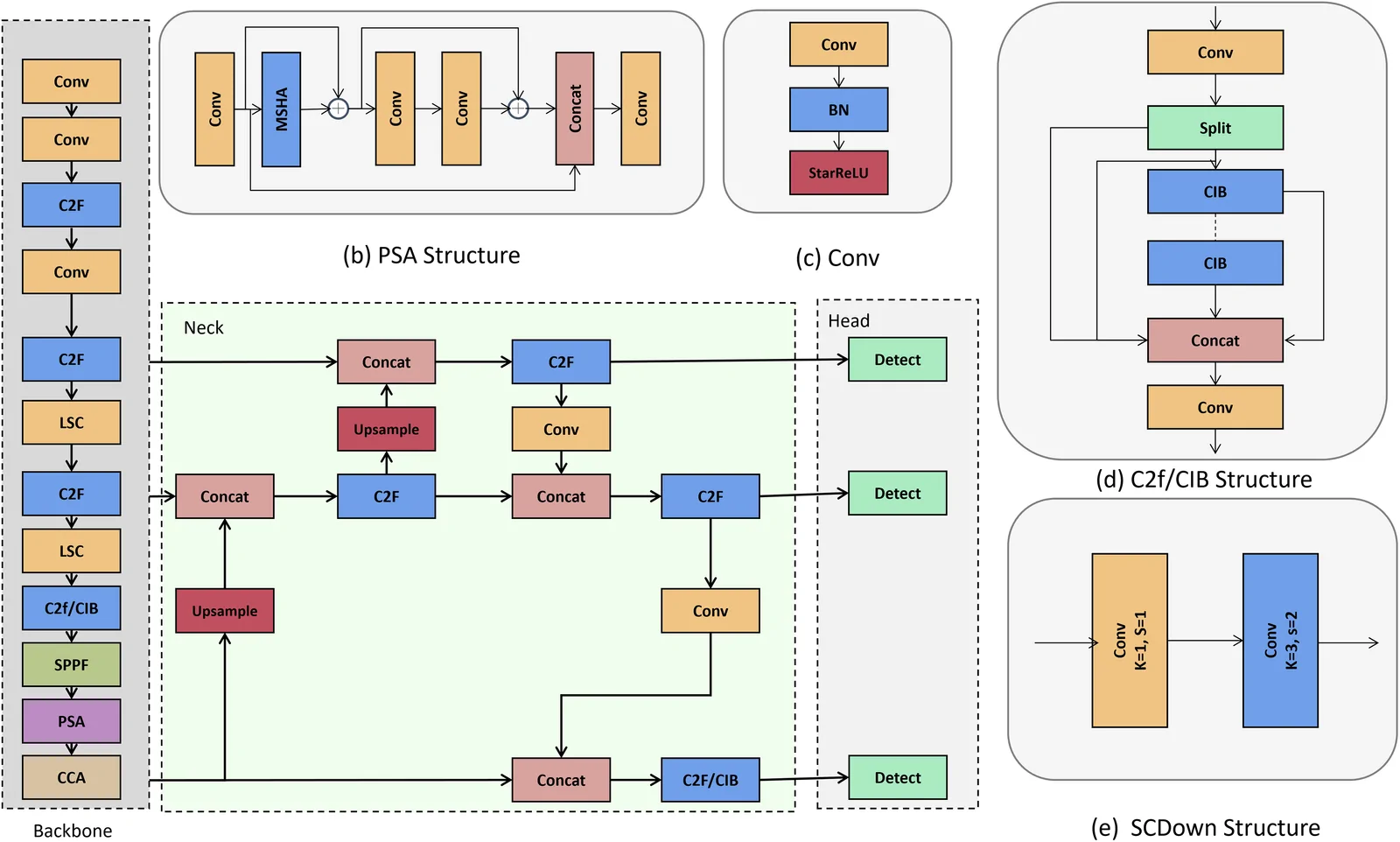
Overall Network Architecture.

This paper employs the lightweight detector YOLOv10n as the baseline to construct CAS-YOLO, an object detection network tailored for civilian infrared UAV scenarios, with its overall topological structure illustrated in Fig 1(a). The model comprises three principal components: the Backbone, Neck, and Head. To enhance the nonlinear expressive capability of features throughout the network, the activation functions in basic convolutional units are uniformly replaced with StarReLU, as depicted in Fig 1(c).

During feature extraction, input images undergo progressive downsampling and abstraction through the backbone. LSC is selectively placed after the intermediate C2f stages, where spatial detail remains sufficient, to enhance the directional responses of elongated infrared targets while reducing interference from thermal clutter. The resulting features are then passed to CCA at the terminal low-resolution stage for efficient global context aggregation before multi-scale fusion in the neck, forming a sequential pathway from local directional enhancement to global contextual aggregation.

Following feature extraction, the neck network employs bidirectional pathways—top-down and bottom-up—to fuse deep high-level semantic information with shallow high-resolution details through upsampling and channel concatenation operations. Finally, the detection head receives the aggregated multi-scale features and decodes target bounding boxes and category information in parallel through the corresponding Detect modules.

### 3.2. LSC module

Under nadir-view infrared UAV imaging, targets such as vehicles and pedestrians often exhibit strong anisotropy and extreme aspect ratios. Conventional square convolution windows introduce substantial irrelevant thermal-background information when processing such elongated targets, weakening feature discrimination and localization. Accordingly, LSC is deployed at intermediate backbone stages, where spatial detail is still retained, and uses sequential orthogonal large-kernel strip convolutions to enhance directional target structures while limiting interference from irrelevant background regions.

The LSC module achieves efficient modeling of long-range anisotropic context by fusing small-size square convolutions with large-size strip convolutions, without introducing complex parallel branch structures. Given an input feature map $X\in R^{C\times H\times W}$, the module first applies a depthwise separable square convolution with local receptive field to extract fundamental local contextual features and output an intermediate feature map *Z*:


$$\begin{array}{c} Z=\text{DWConv}(X)\; \end{array}$$

**Fig 2:**
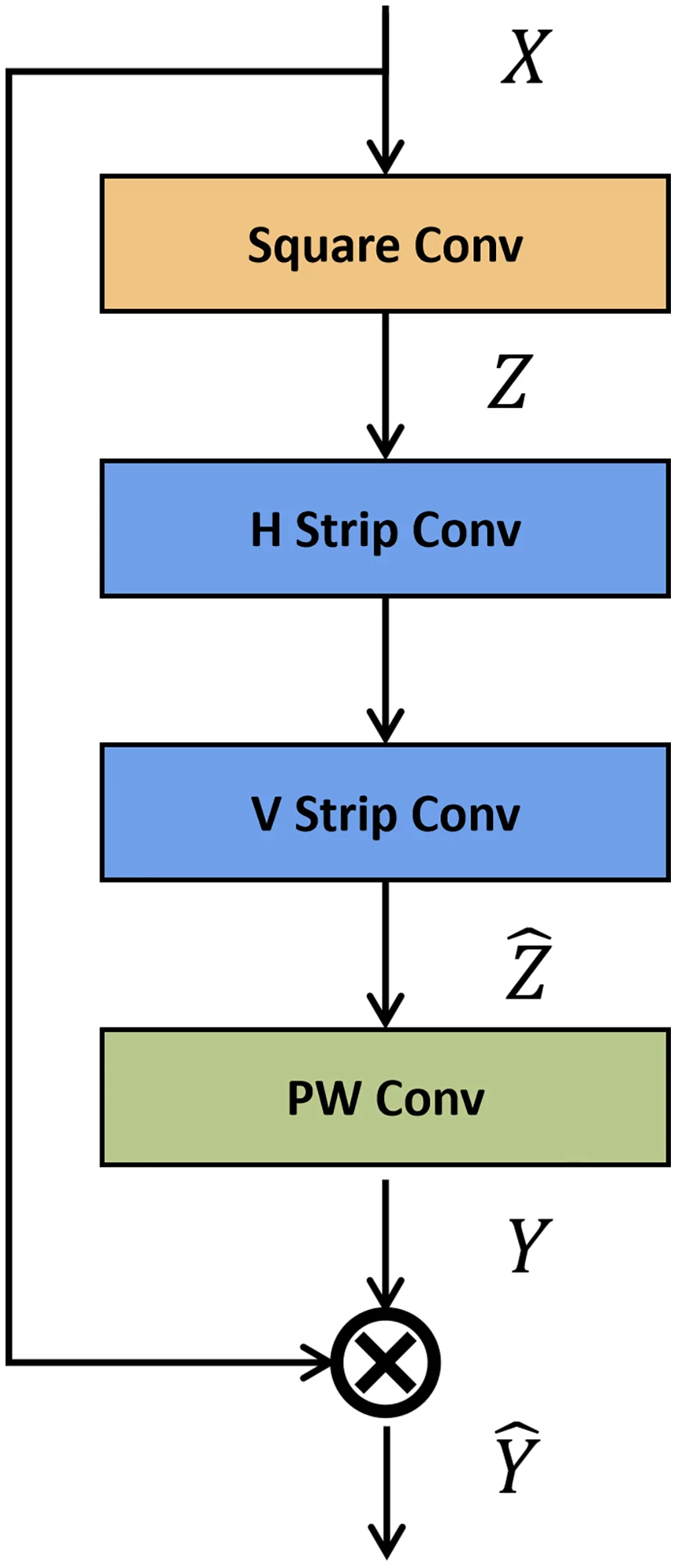
Structure of the LSC module. It combines square and horizontal/vertical strip convolutions to capture local and directional features, followed by pointwise convolution for adaptive feature enhancement.


To further capture long-range spatial dependencies of high-aspect-ratio targets, the network consecutively applies two orthogonal depthwise separable LSC upon Z. Unlike conventional convolutions that extract features simultaneously across both spatial dimensions, LSC enables the network to explicitly focus feature responses along a single horizontal or vertical axis. Let the horizontal and vertical strip convolution kernel sizes be 1 × *K* and *K* × 1, respectively; the spatial feature aggregation at this stage can be formulated as (Fig 2):

$$\begin{array}{c} \hat{Z}={\text{DWConv}}_{K\times \text{1}}({\text{DWConv}}_{\text{1}\times K}(Z)) \end{array}$$

In the final CAS-YOLO configuration, the strip-kernel length is set to K=19 based on the controlled comparison reported in Table 6.

After obtaining anisotropic features containing long-range dependencies, the LSC module performs pointwise convolution on feature map *Z* to facilitate cross-channel interactions and generate a spatial attention weight map *Y*. Each spatial position in this weight map encodes feature aggregation information within an extensive receptive field. Finally, the attention weight map *Y* is multiplied element-wise with the original input feature map *X* to achieve adaptive reweighting of the input features:


$$\begin{array}{c} \hat{Y}=X⊙Y \end{array}$$

where ⊙ denotes element-wise multiplication and $\hat{Y}$ represents the final output of the module. Without introducing additional spatial or channel attention operators, the LSC module combines local square convolutions with large-receptive-field strip convolutions to enhance directional feature extraction for elongated infrared targets.

### 3.3. Circulant contextual attention mechanism

**Fig 3:**
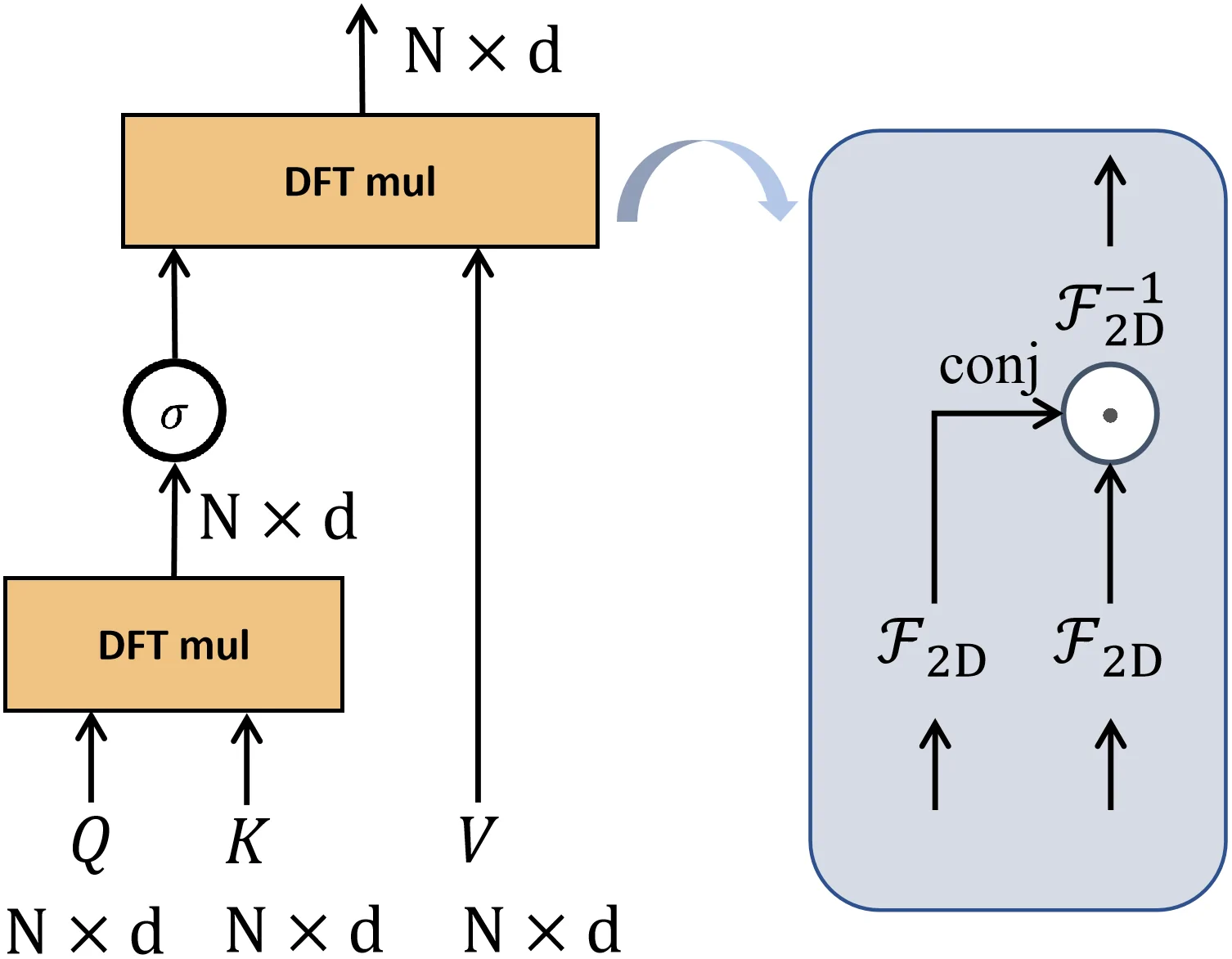
Structure of the CCA module. It models interactions among queries, keys, and values in the frequency domain using DFT, enabling efficient global context modeling.

In CAS-YOLO, CCA is placed after the terminal PSA block at the lowest-resolution backbone stage, where the feature map has lower spatial resolution and higher-level semantics. This placement supports global context modeling for distinguishing faint infrared targets from cluttered thermal backgrounds while avoiding the cost of applying attention to higher-resolution features. CCA exploits the tendency of self-attention matrices to approach block-circulant matrices with circulant blocks (BCCB). Instead of directly computing the dense attention matrix $A=QK^{⊤}/\sqrt{d}$, it projects the matrix onto the nearest BCCB subspace. Because multiplication between BCCB and feature matrices is equivalent to two-dimensional circular cross-correlation, CCA transfers the operation to the frequency domain through the two-dimensional discrete Fourier transform (Fig 3).

Specifically, given queries *Q*, keys *K*, and values $V\in R^{N\times d}$ (where *d* denotes the attention head dimension), CCA first computes the base kernel a of the BCCB attention matrix in the frequency domain:


$$\begin{array}{c} a=\frac{\text{1}}{N\sqrt{d}}F_{\text{2}D}^{-\text{1}}\left(\overset{\;―}{F_{\text{2}D}(Q)}⊙F_{\text{2}D}(K)\right)\cdot 1_{d\times \text{1}} \end{array}$$

where $F_{\text{2}D}$ and $F_{\text{2}D}^{-\text{1}}$ represent the two-dimensional discrete Fourier transform and its inverse, respectively; ⊙ denotes the Hadamard product; (⋅) indicates complex conjugate operation; and 1_d×1_ serves for implicit summation across channel dimensions. After obtaining the base kernel a and applying *σ* (Softmax function) normalization, the final global context aggregation feature O is similarly computed through frequency-domain multiplication:


$$\begin{array}{c} O=F_{\text{2}D}^{-\text{1}}\left(\overset{\;―}{F_{\text{2}D}(\sigma (a))}⊙F_{\text{2}D}(V)\right) \end{array}$$

Furthermore, the strict BCCB structure forces both row and column sums of the normalized attention matrix to equal 1, which attenuates the model's focusing capability on salient infrared objects. To address this, CCA introduces an input-dependent dynamic reweighting mechanism at the output stage:


$$\begin{array}{c} O_{final}=O⊙\text{SiLU}(XW_{T}) \end{array}$$

where $W_{T}\in R^{C\times d}$ represents learnable weights. This design compensates for the performance degradation induced by structural constraints, enabling the model to adaptively enhance feature responses of critical infrared objects.

### 3.4. Application of the StarReLU activation function

In lightweight detection networks for civilian infrared UAV applications, the computational and power constraints of edge devices limit the use of expensive nonlinear operators. Infrared images typically contain weak texture, low target-background contrast, and complex thermal noise, which place greater demands on nonlinear feature transformation. Meanwhile, increasing activation complexity throughout the network can directly raise computational overhead. To balance representation capability and efficiency, CAS-YOLO uniformly applies the lightweight StarReLU activation to its basic convolutional units. Its quadratic response and learnable scaling and bias terms provide an adjustable nonlinear transformation with limited additional computation. Its mathematical formulation is defined as:


$$\begin{array}{c} StarReLU(x)=s\cdot (max(\text{0},x){)}^{\text{2}}+b \end{array}$$

where s denotes a learnable scaling coefficient and b denotes a learnable bias. For $\begin{array}{c} x\geq \text{0} \end{array}$, StarReLU produces a learnable quadratic response, while s and b adjust the activation scale and offset during training. Its derivative is given by:


$$\begin{array}{c} \frac{d}{dx}StarReLU(x)=\left\{ \begin{array}{cccc} & \text{0}\; & & x<\text{0} \end{array}\right. \end{array}$$

The derivative is zero for negative inputs and increases linearly with x for non-negative inputs, with its magnitude scaled by s This formulation provides a learnable nonlinear response while retaining simple element-wise computation.

## 4. Experiments

### 4.1. Datasets and evaluation metrics

All experiments use the publicly available HIT-UAV dataset [17] as the primary UAV-based benchmark, while the vehicle-mounted FLIR thermal dataset [18] is used only for auxiliary cross-domain evaluation. HIT-UAV is designed for UAV object detection in nighttime and low-visibility environments, with collection scenes including schools, parking lots, roads, and playgrounds. The HIT-UAV dataset comprises 2898 high-resolution infrared thermal images (640 × 512 resolution) captured by UAVs equipped with FLIR long-wave infrared thermal imagers. The dataset contains 24899 manually annotated target instances across five categories: Person, Car, Bicycle, Other Vehicle, and DontCare. According to the official HIT-UAV definition, DontCare denotes ambiguous objects without reliable semantic labels; therefore, mAP is calculated only over Person, Car, Bicycle, and OtherVehicle, while all images and the official dataset split are retained. To ensure fairness and reproducibility of model evaluation, this paper strictly adheres to the official partitioning protocol of the dataset, dividing all data into a training set of 2029 images, a validation set of 290 images, and a test set of 579 images.

To complement the primary evaluation on HIT-UAV, the FLIR-ADAS dataset [18] is used as an auxiliary cross-domain dataset. It contains ground-level forward-looking thermal images collected for ADAS and autonomous-driving research and therefore does not reproduce UAV nadir-view imaging conditions. Its use in this study is limited to examining model behavior under one distinct infrared data distribution involving changes in acquisition platform, viewpoint, and scene composition. Following the dataset partition adopted in [18], this study uses 8,862 training images and 1,366 test images with a resolution of 640 × 512.

To objectively and quantitatively evaluate the performance of the proposed CAS-YOLO model on infrared UAV target detection tasks, this paper adopts standard evaluation metrics widely employed in the object detection domain, specifically including Precision, Recall, Average Precision (AP), and mean Average Precision (mAP). Prior to defining these core metrics, three fundamental statistics from the confusion matrix are first introduced: True Positives (TP) represent the number of samples correctly predicted by the model as belonging to the ground-truth target category; False Positives (FP) denote the number of background or non-target instances erroneously predicted as target categories; and False Negatives (FN) indicate the number of ground-truth annotated targets that remain undetected by the model. During evaluation, an Intersection over Union (IoU) threshold is typically set to determine whether predicted bounding boxes successfully match ground-truth bounding boxes, thereby partitioning the aforementioned statistics.

Precision is defined as the proportion of true positives among all samples predicted as positive by the model, emphasizing the model's capability to suppress false detections induced by complex background thermal noise. The formula is:


$$\begin{array}{c} Precision=\frac{TP}{TP+FP} \end{array}$$

Recall is defined as the proportion of true positives correctly predicted by the model among all actual positive samples in the test set, emphasizing the model's capability to capture faint potential objects without omission. The formula is:


$$\begin{array}{c} \text{Recall}=\frac{TP}{TP+FN}, \end{array}$$

AP is essentially the area enclosed by the Precision-Recall curve (*P-R* curve) and the coordinate axes. By continuously varying the model's confidence threshold, a series of corresponding precision and recall values are computed to plot the *P-R* curve. For a specific target category, its average precision can be expressed as the integral of precision *P* with respect to recall *R*:


$$\begin{array}{c} AP=\int_{\text{0}}^{\text{1}}\;P(R)dR \end{array}$$

mAP is the arithmetic mean of AP values across all independent target categories, effectively eliminating evaluation bias induced by imbalanced data volumes in single categories. Assuming the dataset contains C target categories with category-wise average precision denoted as $AP_{i}$, the mAP is calculated as:


$$\begin{array}{c} mAP=\frac{\text{1}}{C}\sum_{i=\text{1}}^{C}\;AP_{i} \end{array}$$

In subsequent experimental comparisons, this paper primarily employs mean Average Precision at an IoU threshold of 0.5 (i.e., mAP@0.5) as the principal metric for measuring performance differences among algorithms, thereby comprehensively validating the effectiveness of the CAS-YOLO model for multi-scale, multi-morphology target detection under infrared UAV perspectives.

### 4.2. Implementation details

The proposed model is implemented based on the PyTorch deep learning framework, with the experimental environment employing Python 3.8, and training and testing conducted on a platform equipped with an NVIDIA RTX 5090 graphics card (32 GB video memory). To enhance model generalization, random horizontal flipping and random rotation data augmentation strategies are applied to input samples during the training phase. Regarding network hyperparameter settings, input images are uniformly resized to 640 × 640 resolution, with a batch size of 16 and a total of 500 training epochs. Stochastic Gradient Descent (SGD) is employed for parameter optimization, with momentum coefficient and weight decay factor set to 0.937 and 0.0005, respectively. To ensure stable gradient descent and smooth convergence during training, the learning rate adopts a Linear Decay strategy, progressively decreasing from an initial value of 0.01 to a final value of 0.001. All comparison models, including YOLOv10n, were retrained and evaluated using the same dataset split, input resolution, batch size, optimizer, and training epochs.

### 4.3. Comparison to state-of-the-art methods

To evaluate the effectiveness of CAS-YOLO for infrared UAV object detection, this section compares it with representative detection algorithms on the HIT-UAV and FLIR datasets. The evaluated methods include the two-stage Faster R-CNN [19], the Transformer-based RT-DETR [20], lightweight YOLO variants including YOLOv5n [21], YOLOv6n [22], YOLOv8n [23], and YOLOv10n [24], as well as the remote-sensing-specific LSKNet-S [4], PKINet-S [5], and Strip R-CNN-S [1]. All models are evaluated using the same software and hardware environment, dataset partitions, training settings, and evaluation protocol. The category-wise $AP_{\text{0}.\text{5}}$ and overall $mAP_{\text{0}.\text{5}}$ results are reported in Tables 1 and 2.

**Table 1:** Quantitative comparison results of various state-of-the-art object detection algorithms on the HIT-UAV Dataset.

Models	$AP_{0.5}^{Person}$	$AP_{0.5}^{Bicycle}$	$AP_{0.5}^{Car}$	$AP_{0.5}^{OtherVehicle}$	$mAP_{0.5}/\%$
Faster R-CNN	84.2	82.1	94.0	38.4	74.7
RT-DETR	85.3	82.6	93.8	39.0	75.2
YOLOv5n	90.3	90.4	98.2	68.5	86.9
YOLOv6n	88.0	85.7	97.7	65.7	84.3
YOLOv8n	91.9	89.1	98.0	70.0	87.3
YOLOv10n	91.0	90.1	97.9	72.3	87.8
LSKNet-S	90.3	89.4	97.6	70.2	86.9
PKINet-S	90.5	89.6	97.8	70.8	87.2
Strip R-CNN-S	90.6	90.1	98.0	71.7	87.6
Ours	91.5	91.6	98.6	75.2	89.2

**Table 2:** Quantitative comparison results of various state-of-the-art object detection algorithms on the FLIR Dataset.

Models	$AP_{0.5}^{Person}$	$AP_{0.5}^{Bicycle}$	$AP_{0.5}^{Car}$	$mAP_{0.5}/\%$
Faster R-CNN	76.5	72.8	88.7	79.3
RT-DETR	83.1	73.3	88.4	81.6
YOLOv5n	87.8	77.2	91.3	85.4
YOLOv6n	88.7	76.6	91.8	85.7
YOLOv8n	88.4	76.9	91.9	85.7
YOLOv10n	87.6	76.6	91.2	85.1
LSKNet-S	86.9	74.9	91.7	84.5
PKINet-S	87.2	75.1	92.3	84.9
Strip R-CNN-S	87.3	75.6	92.2	85.0
Ours	89.2	78.1	92.6	86.6

On HIT-UAV, CAS-YOLO achieves the highest $mAP_{\text{0}.\text{5}}$ of 89.2%, improving upon YOLOv10n by 1.4 percentage points and the strongest remote-sensing-specific comparator, Strip R-CNN-S, by 1.6 percentage points. CAS-YOLO obtains the highest $AP_{\text{0}.\text{5}}$ values of 98.6% and 91.6% for Car and Bicycle, respectively. For OtherVehicle, which frequently exhibits elongated shapes and is easily confused with thermal background clutter, its $AP_{\text{0}.\text{5}}$ reaches 75.2%, representing a 2.9-point improvement over YOLOv10n. Together with the ablation results, these improvements support the effectiveness of directional structure enhancement and deep contextual aggregation for challenging infrared targets. The model also achieves a competitive $AP_{\text{0}.\text{5}}$ of 91.5% for Person.

On the ground-level FLIR dataset, CAS-YOLO achieves an $mAP_{\text{0}.\text{5}}$ of 86.6%, exceeding YOLOv10n and Strip R-CNN-S by 1.5 and 1.6 percentage points, respectively. It also obtains the highest $AP_{\text{0}.\text{5}}$ values for Person, Bicycle, and Car. Because FLIR differs from HIT-UAV in acquisition platform, viewpoint, and scene distribution, these results are used only as auxiliary evidence under one ground-level infrared setting and are not interpreted as UAV cross-view generalization.

**Fig 4:**
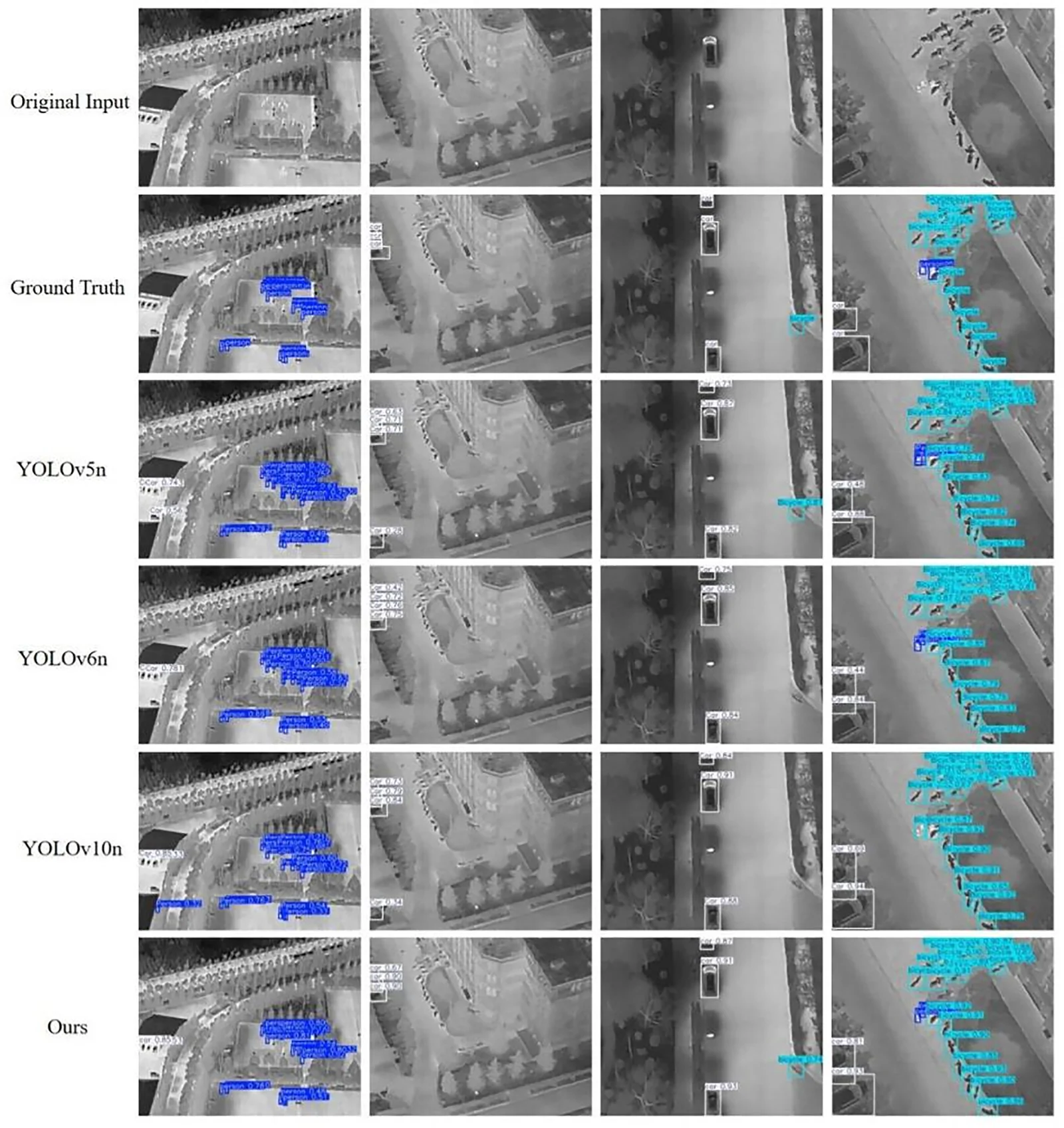
Comparison of our model with state-of-the-art models.

To more intuitively demonstrate the detection efficacy of the proposed CAS-YOLO model in actual complex infrared environments, this section selects representative challenging scenarios from the HIT-UAV dataset for qualitative visual comparison. As illustrated in Fig 4, comparison scenarios extensively cover densely overlapping pedestrians and bicycles, extremely low-contrast faint vehicles, and street regions severely disturbed by thermal background noise. From the prediction results of mainstream state-of-the-art algorithms in the figure, it is evident that under objective conditions where high-altitude infrared images lack color-texture cues and target pixels are extremely sparse, conventional algorithms universally face severe missed detection and false detection bottlenecks. In dense pedestrian and bicycle scenarios, comparison algorithms struggle to effectively disentangle mutually occluded instance boundaries, resulting in numerous elongated targets being ignored or assigned low confidence; when vehicles undergo thermodynamic confusion with asphalt surfaces, comparison networks are highly susceptible to interference from local thermal spot noise, producing localization boundary offsets or direct false-positive predictions.

**Fig 5:**
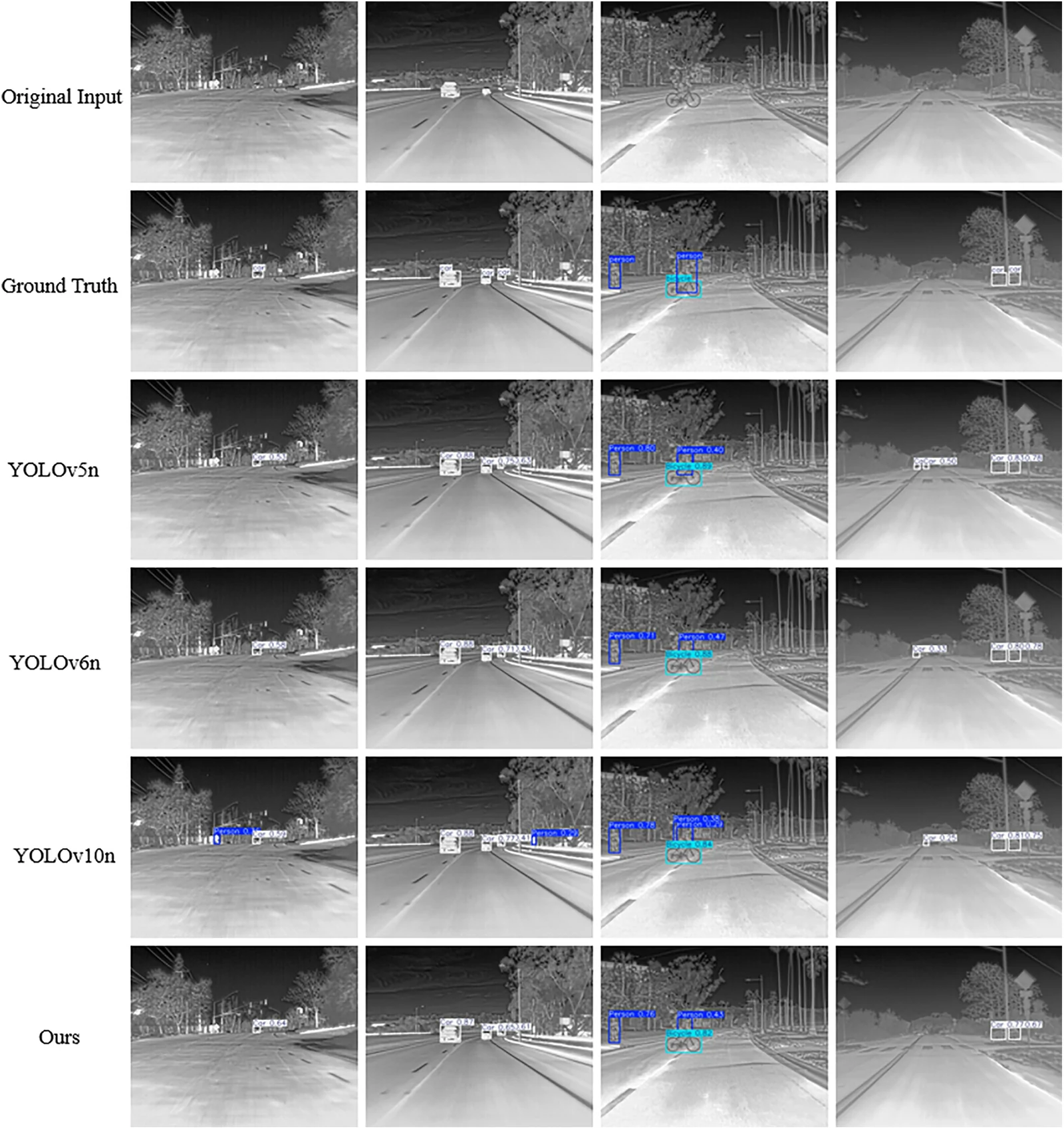
Comparison of our model and state-of-the-art models on the FLIR dataset.

Fig 5 further presents qualitative results on the vehicle-mounted FLIR dataset to examine the behavior of different models under a distinct acquisition platform and scene distribution. Fundamentally distinct from UAV high-altitude nadir perspectives, onboard viewpoints confront extreme scale variations and severe foreground occlusion challenges. Observing Fig 5, when processing distant low-contrast vehicles and nearby overlapping pedestrians and bicycles, the comparison models frequently exhibit missed detections or inaccurate overlapping bounding boxes. In comparison, CAS-YOLO produces more complete detections and fewer visible localization errors. These qualitative results are consistent with the quantitative results in Table 2 and provide additional support for the effectiveness of CAS-YOLO in the evaluated ground-level FLIR scenes.

In the illustrated HIT-UAV scenes, CAS-YOLO produces fewer missed detections and more accurate bounding boxes for dense, elongated, and low-contrast targets than the displayed comparison models. These observations are consistent with the category-wise improvements reported in Table 1 and the controlled ablations in Tables 3–7, supporting the roles of directional structure enhancement and deep contextual aggregation in the evaluated architecture.

### 4.4. Ablation studies

To rigorously validate the independent contributions and synergistic effects of each proposed module, this section conducts systematic ablation experiments on the HIT-UAV dataset. Table 3 evaluates the independent and joint effects of LSC and CCA on HIT-UAV, while Table 4 reports the corresponding results on FLIR. StarReLU is retained in these configurations and is separately evaluated against alternative activation functions in Table 5.

**Table 3:** Results of ablation experiments for different core modules on the HIT-UAV dataset.

Baseline	LSC	CCA	HIT-UAV				
			$AP_{0.5}^{Person}$	$AP_{0.5}^{Bicycle}$	$AP_{0.5}^{Car}$	$AP_{0.5}^{OtherVehicle}$	$mAP_{0.5}/\%$
√			91.1	90.3	97.9	72.4	87.9 ± 0.14
√	√		91.3	90.9	98.3	74.6	88.8 ± 0.12
√		√	91.4	91.4	98.3	74.8	89.0 ± 0.13
√	√	√	91.5	91.6	98.6	75.2	89.2 ± 0.12

**Table 4:** Ablation experiment results for different core modules on the FLIR dataset.

Baseline	LSC	CCA	FLIR			
			$AP_{0.5}^{Person}$	$AP_{0.5}^{Bicycle}$	$AP_{0.5}^{Car}$	$mAP_{0.5}/\%$
√			87.8	76.6	91.4	85.3 ± 0.22
√	√		88.4	77.1	91.9	85.8 ± 0.19
√		√	88.9	77.8	92.3	86.3 ± 0.18
√	√	√	89.2	78.1	92.6	86.6 ± 0.20

**Table 5:** Ablation results for different activation functions on the HIT-UAV dataset.

Activation function	$mAP_{0.5}$ (%, mean ± std)	$\Delta mAP_{0.5}$ (pp)
**StarReLU**	**89.2 ± 0.12**	**–**
SiLU	89.1 ± 0.14	−0.1
HardSwish	89.1 ± 0.18	−0.1
ReLU6	89.0 ± 0.16	−0.2

The class-wise AP values are reported as the means of the same three runs, while their standard deviations are omitted for compactness. To evaluate the relative contributions and complementary effects of LSC and CCA, controlled ablation experiments are conducted on the HIT-UAV and FLIR datasets. Each configuration is independently evaluated over three runs using different random seeds, and mAP₀.₅ is reported as mean ± standard deviation. To isolate the effects of LSC and CCA, StarReLU is retained in all configurations. Therefore, the baseline in Tables 3 and 4 denotes YOLOv10n using StarReLU but without LSC or CCA. This also explains the slight difference between 87.9 ± 0.14% in Tables 3 and 87.8% obtained by the original YOLOv10n in Table 1.

On HIT-UAV, incorporating LSC or CCA increases mAP_0.5_ from 87.9 ± 0.14% to 88.8 ± 0.12% and 89.0 ± 0.13%, respectively, while combining both components further improves it to 89.2 ± 0.12%. LSC particularly improves the representation of elongated targets in the Other Vehicle category, whereas CCA further benefits the Bicycle and Other Vehicle categories. On FLIR, mAP₀.₅ similarly increases from 85.3 ± 0.22% to 85.8 ± 0.19%, 86.3 ± 0.18%, and 86.6 ± 0.20%. The consistent improvement trends and limited run-to-run variations across both datasets provide further support for the effectiveness and complementary contributions of LSC and CCA.

To further examine the selection of the activation function, StarReLU in the complete CAS-YOLO architecture is replaced with SiLU, HardSwish, and ReLU6 while all other settings are kept unchanged. Each configuration is independently evaluated over three runs, and the results are reported as mean ± standard deviation in Table 5.

As shown in Table 5, StarReLU achieves the highest mean $mAP_{\text{0}.\text{5}}$ of 89.2% and the lowest standard deviation of 0.12. SiLU and HardSwish are both 0.1 percentage points lower, while ReLU6 is 0.2 percentage points lower. Although the differences are modest, StarReLU provides the best mean accuracy and run-to-run stability among the evaluated activation functions, supporting its use in the final CAS-YOLO configuration. We further examine the sensitivity of CAS-YOLO to the LSC kernel size and the token-reweighting position in CCA, with all other settings kept unchanged. Since the LSC kernel size and CCA reweighting strategy are particularly relevant to the representation of anisotropic targets, $AP_{\text{0}.\text{5}}^{OtherVehicle}$ is additionally reported in Tables 6 and 7, together with the overall $mAP_{\text{0}.\text{5}}$.

**Table 6:** Ablation results for different LSC kernel sizes on the HIT-UAV dataset.

Kernel size K	$AP_{0.5}^{OtherVehicle}$ (%)	$mAP_{0.5}$ (%, mean ± std)	$\Delta mAP_{0.5}$ vs. K = 19 (pp)
11	74.3	88.7 ± 0.16	−0.5
15	74.8	89.0 ± 0.14	−0.2
**19**	**75.2**	**89.2 ± 0.12**	**–**
23	74.6	88.9 ± 0.15	−0.3

**Table 7:** Ablation results for different token-reweighting strategies in CCA on the HIT-UAV dataset.

CCA configuration	$AP_{0.5}^{OtherVehicle}$ (%)	$mAP_{0.5}$ (%, mean ± std)	$\Delta mAP_{0.5}$ vs. post-reweighting (pp)
Without reweighting	74.5	88.8 ± 0.17	−0.4
Pre-reweighting	74.9	89.0 ± 0.14	−0.2
**Post-reweighting**	**75.2**	**89.2 ± 0.12**	**–**

As shown in Table 6, K = 19 achieves the highest $mAP_{\text{0}.\text{5}}$ of 89.2% and the highest $AP_{\text{0}.\text{5}}^{OtherVehicle}$ of 75.2% among the evaluated kernel sizes. Smaller kernels provide insufficient directional context, whereas increasing K to 23 may introduce additional background interference. Table 7 shows that post-reweighting achieves an $mAP_{\text{0}.\text{5}}$ of 89.2%, compared with 88.8% without reweighting and 89.0% with pre-reweighting. It also produces the highest $AP_{\text{0}.\text{5}}^{OtherVehicle}$ of 75.2%, indicating that applying token reweighting after contextual aggregation provides more effective feature calibration. These results support the selection of K = 19 and post-reweighting within the evaluated configurations.

## 5. Conclusion

To address thermal infrared object detection in low-visibility and cluttered civilian UAV environments, this paper presents CAS-YOLO, an efficient lightweight architecture based on YOLOv10n for resource-constrained deployment. CAS-YOLO organizes its components according to the characteristics of infrared imagery and the computational profiles of different network stages. LSC is selectively placed at intermediate backbone stages, where sufficient spatial detail is retained, to enhance the directional structures of small and elongated targets while reducing interference from surrounding thermal clutter. The resulting features are subsequently passed to CCA at the terminal low-resolution stage to aggregate long-range contextual information for distinguishing faint targets from complex backgrounds without applying global modeling to high-resolution feature maps. StarReLU is further deployed throughout the basic convolutional units to provide lightweight nonlinear transformation for weak-texture infrared features. Together, these components establish a sequential pathway from local directional structure enhancement to deep global context aggregation and are systematically integrated with the multi-scale neck and detection head, enabling CAS-YOLO to balance feature representation and computational efficiency.

Quantitative and qualitative evaluations on HIT-UAV, together with auxiliary evaluation on the ground-level FLIR dataset, show that CAS-YOLO improves detection accuracy under the reported experimental settings. The repeated-run ablations support the individual effects of the three components and the complementary effects of LSC and CCA. FLIR does not replace validation across diverse UAV-specific conditions. Future work will evaluate the model across different flight altitudes, nighttime and multi-illumination conditions, and adverse weather, and will measure FPS, inference latency, memory usage, and power consumption on representative embedded platforms.
